# Conservative Management of Maxillary and Mandibular Fractures in a Pediatric Patient With a Modified Open Cap Splint: A Case Report

**DOI:** 10.7759/cureus.55191

**Published:** 2024-02-28

**Authors:** Rutuja Ragit, Punit R Fulzele, Sanjana N Wadewale, Nitin Bhola, Dhruvi R Solanki, Nilima R Thosar

**Affiliations:** 1 Pediatric and Preventive Dentistry, Sharad Pawar Dental College, Datta Meghe Institute of Higher Education and Research, Wardha, IND; 2 Oral and Maxillofacial Surgery, Sharad Pawar Dental College, Datta Meghe Institute of Higher Education and Research, Wardha, IND; 3 Pediatric and Preventive Dentistry, Sharard Pawar Dental College, Datta Meghe Institute of Higher Education and Research, Wardha, IND

**Keywords:** case report, general anaesthesia, circum-mandibular wiring, circumzygomatic wiring, pediatric dental trauma, paediatric facial fracture, le fort fracture, mandibular fracture, maxillary fracture, cap splint

## Abstract

Pediatric maxillofacial fractures, which are not very prevalent, account for around 5% of all face injuries. Children under the age of 13 are more susceptible to craniofacial injuries because they have a larger cerebral mass-to-body ratio than adults. The fracture pattern in children does not resemble that of adults, due to which the treatment of pediatric fractures differs from that of adults and can pose substantial difficulties to the pediatric dentist due to many factors, including the complex anatomy of the developing jaw. In this case report, a 5-year-old male patient presented with an injury to the upper and lower jaw. A case was managed with a conservative approach by using a modified open cap splint. A radiographic investigation, including CT brain and face, was done, which revealed the mandibular symphyseal fracture, bilateral condyle, and right Lefort II fracture. A modified open cap splint was fabricated and fixed with circummandibular and circumzygomatic wiring under general anesthesia. After two months, the fractured site showed good healing on orthopantomography (OPG), and satisfactory occlusion was achieved. The patient was kept on monthly follow-ups for up to five months. Treatment guidelines for pediatric maxillary and mandibular fractures are different from those for adults in that most pediatric cases are managed by a conservative approach. Cap splints are a versatile treatment option for juvenile mandibular fractures because they can be used to restore function and aesthetics with minimal morbidity, do not impede jaw growth or the development of dentition, and can be applied to patients of a wider range of ages.

## Introduction

In comparison to fractures in the adult population, pediatric fractures in the maxillofacial region are thought to comprise 5% of all facial fractures, among which mandibular fractures represent 56% of all facial injuries. For children under the age of 16, the occurrence of pediatric facial fractures ranges from 1% to 14%, whereas for children under the age of 5, it varies from 0.87% to 1% [1]. The most frequent causes of facial fractures in patients under the age of five are falls (44%), car accidents (25%), and interpersonal aggression (25%), with variance due to social, cultural, and environmental factors [2]. Boys are more likely to experience trauma than females since they tend to be involved in sports and physical activities more than girls. As age increases, fracture patterns also alter [3].

In pediatric patients, Le Fort fractures are uncommon, and managing them becomes more difficult as the craniofacial musculoskeletal system and dentition are still developing. Because of the mandible's resilience and the existence of tooth buds that serve as tiny anchors to hold the unit together, the majority of pediatric mandibular fractures are nondisplaced [3]. Treatment methods need to be modified according to the child's stage of growth, anatomy, physiology, and psychology at the time of the trauma. Therefore, except for severely displaced fracture segments, closed reduction is the preferred choice of treatment [2].

Treatment guidelines for pediatric maxillary and mandibular fractures differ from those for adults in that most pediatric cases are managed by a conservative approach. The standard internal fixation and open reduction treatment modalities are less applicable in pediatric patients [4]. Cap splints, circumferential wiring, open reduction, resorbable plates, and modified orthodontic brackets are some of the treatment modalities available for treating mandibular fractures in children [5]. Cap splints are a versatile treatment option for juvenile mandibular fractures because they can be used to restore function and aesthetics with minimal morbidity, do not impede jaw growth or the development of dentition, and can be applied to patients of a wider range of ages [1]. This case report describes the conservative management of a five-year-old male patient with a mandibular symphyseal fracture, bilateral condyle fractures, and a right Le Fort II fracture. The treatment involved the use of a modified open cap splint, incorporating arch bar hooks and molar tubes for intermaxillary fixation, along with circum-zygomatic wiring and circum-mandibular wiring.

## Case presentation

A five-year-old male patient presented to the Department of Oral and Maxillofacial Surgery and the Department of Pediatric and Preventive Dentistry with an injury to his upper and lower jaw four days ago. A history of alleged falls from the first floor while playing was given by the patient’s father. No history of vomiting, seizures, or loss of consciousness following trauma was given. The patient visited a private hospital in Amravati, from where he was referred to the pediatric dentistry department.

**Figure 1:**
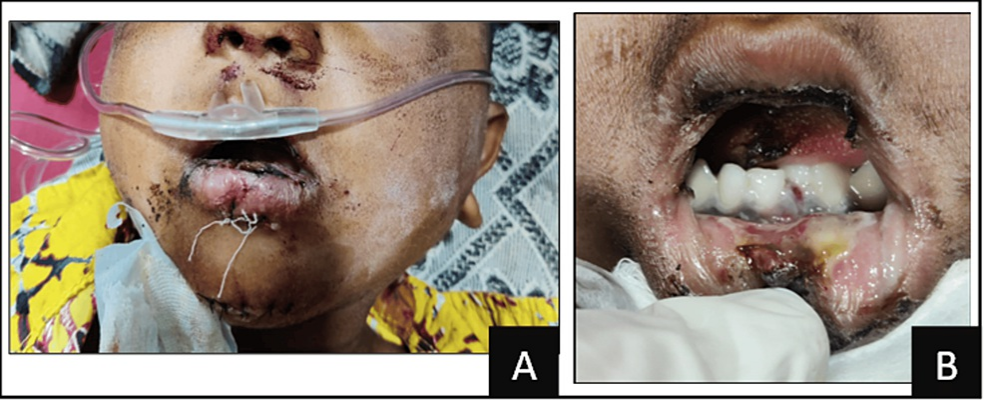
(A) Preoperative extraoral image; (B) preoperative intraoral image.

Gross facial asymmetry was noted along with diffuse edema and erythema in the middle and lower third of the face bilaterally. Sublingual hematoma and laceration of the lip were observed. Intra-orally, on examination, swelling was seen below the vermilion border of the lower lip that extended inferiorly to the lingual vestibule. On palpation, a step deformity was noted between the lower primary central incisor, along with tenderness along the lower border of the mandible at the site of the step deformity (Figure 1). The inter-incisal opening was 14 mm. Hard-tissue examination revealed avulsed 51, 61, and 62; also, the posterior teeth were not in occlusion. None of the teeth were mobile, and teeth 54 and 64 were carious.

**Figure 2:**
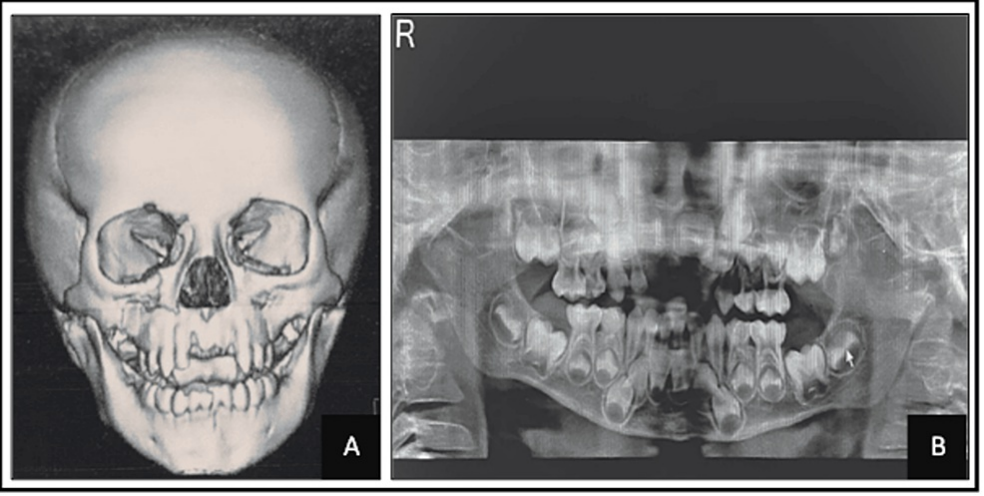
Preoperative intraoral image: (A) CT brain and face; (B) OPG. OPG, orthopantomography; CT, computed tomography

Radiographic investigations were done. Preoperative orthopantomography (OPG) also revealed symphyseal fracture with step deformity along the lower border of the mandible with bilateral condylar fracture and right Le Fort II fracture (Figure 2). CT scans of the brain and face revealed an accumulation of fluid in maxillary sinuses bilaterally with a fracture of the maxilla and a slightly displaced fracture of the medial wall of the right orbit. Also, a bilateral condylar fracture was seen. Symphyseal fracture with 2 mm displacement of the fractured segments was also confirmed radiographically. The clinical and radiographic examination has given a final diagnosis of bilateral condylar fracture, symphyseal fracture, and right Le Fort II fracture.

Treatment

**Figure 3:**
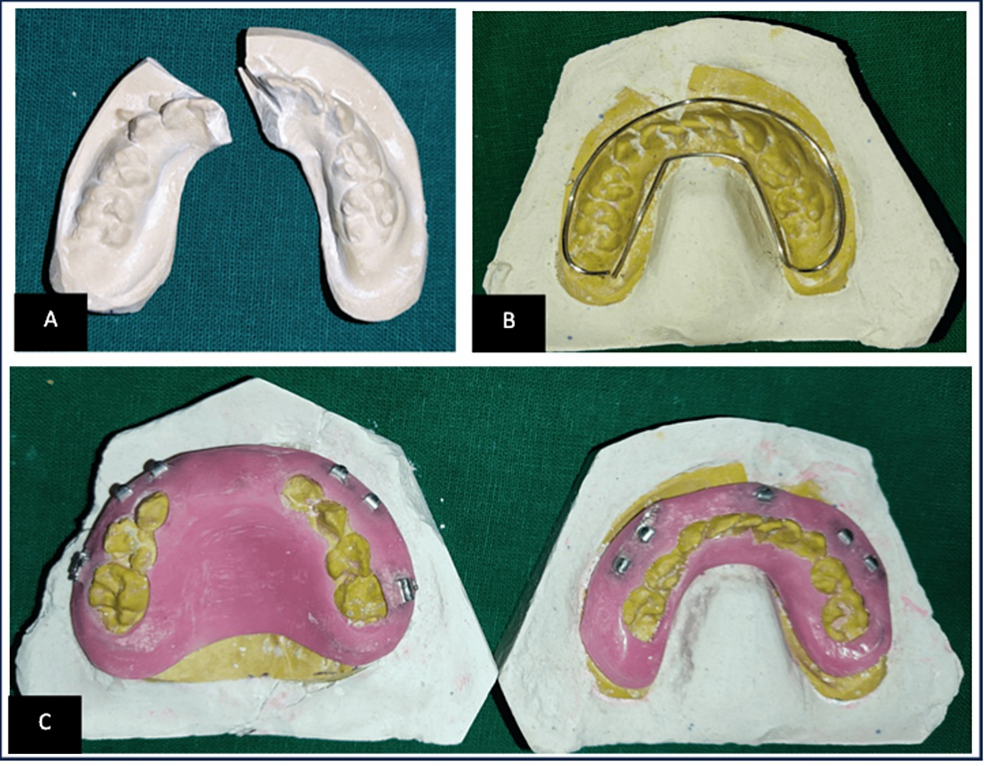
Fabrication of a modified cap splint: (A) mandibular cast split to imitate the fracture; (B) fabrication of a 19-gauge wire; (C) open cap splint fabricated on maxillary and mandibular arches with arch bars.

For the fabrication of the open cap splint, alginate impressions of the upper and lower jaws were taken before surgery. Dental stone type III casts were poured. A 19-gauge wire was used to form a strong framework for an open cap splint, which was secured distal to the deciduous maxillary second molar. Maxillary acrylic splints were then fabricated using cold-cure acrylic resin. In the case of the mandible, there was step formation in the anterior mandible between two central incisors; therefore, to replicate that, the stone cast was split at the site of fracture with a disc bur. The cast segments were brought into normal position and then held in a reduced position. Occlusion with the opposite cast was checked and stabilized with the help of beading wax. In contrast to the cap splint construction on the undisplaced maxillary cast, where no repositioning of the broken parts was necessary, an open cap splint was subsequently made on this newly formed mandibular cast. The occlusal surfaces were left open in both casts, and buccal and lingual flanges were fabricated. A newer approach was taken for occlusal stability and closed reduction wherein during the fabrication of open cap splint, arch bar hooks were incorporated into the acrylic resin for intermaxillary fixation. In maxillary splint, molar tubes were placed in a deciduous second molar region for inter-zygomatic wiring (Figure 3).

Procedure Under General Anesthesia

**Figure 4:**
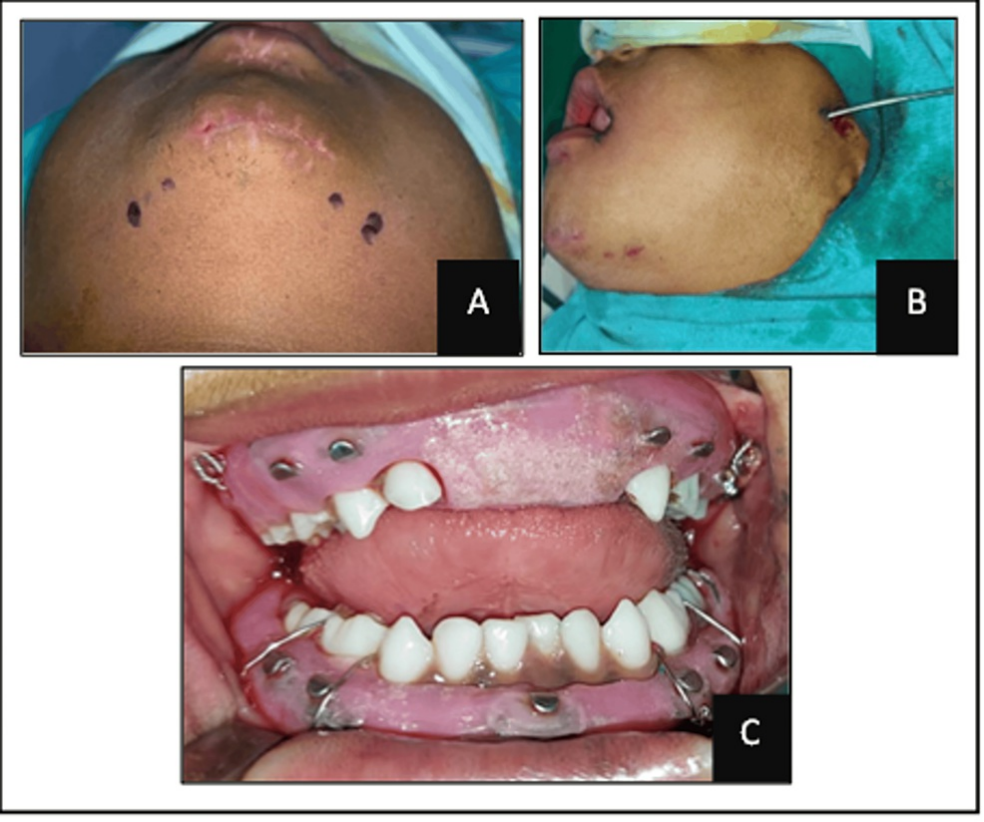
Intraoperative image: (A) marking points for awl insertion for circum-mandibular; (B) insertion of awl for circum-zygomatic wiring; and (C) fixing the maxillary and mandibular cap splints.

**Figure 5:**
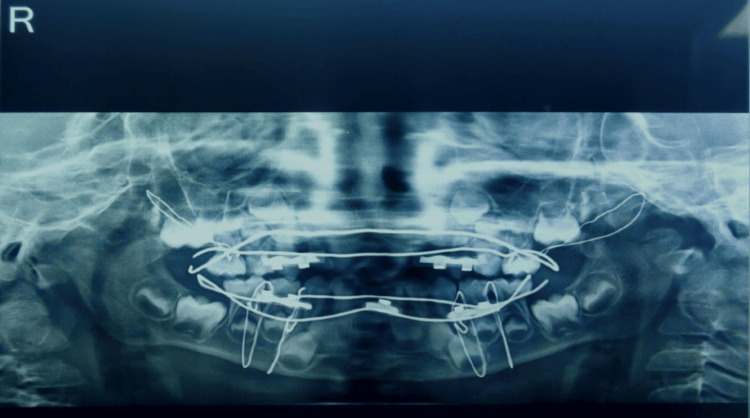
Postoperative panoramic image.

After obtaining aesthetic fitness for surgery, under general anesthesia, segments of the mandible were reduced with the help of digital pressure and the surgical splint. Stab incisions were made bilaterally at the inferior border of the mandible, and a William Kelsey Fry awl was introduced (Figure 4). Circum-mandibular wiring was done to stabilize the open acrylic mandibular cap splint. Subsequently, the maxillary splint was positioned and secured with bilateral circum-zygomatic wiring, with the wires engaging the buccal tubes on the splint. Following surgery, OPG was taken to determine the position of the wires and to assess the degree of fracture reduction in case of displaced fractures (Figure 5). Intermaxillary fixation with elastics was done on the second postoperative day for 10 days. Postoperative instructions included avoiding physical activity, a soft diet, and 0.12% chlorhexidine mouthwash. Antibiotic treatment was instituted for five days.

Follow-Up and Outcome of Interventions

**Figure 6:**
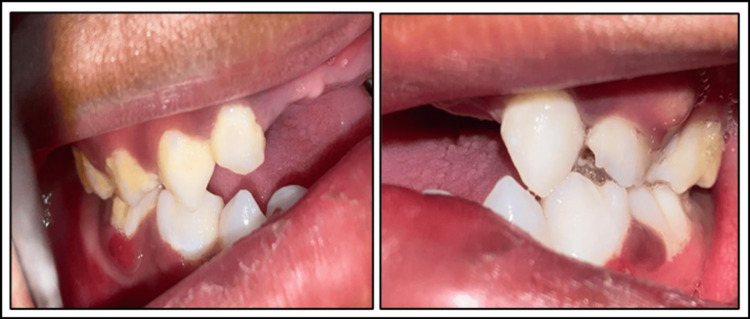
Intra-oral follow-up image.

**Figure 7:**
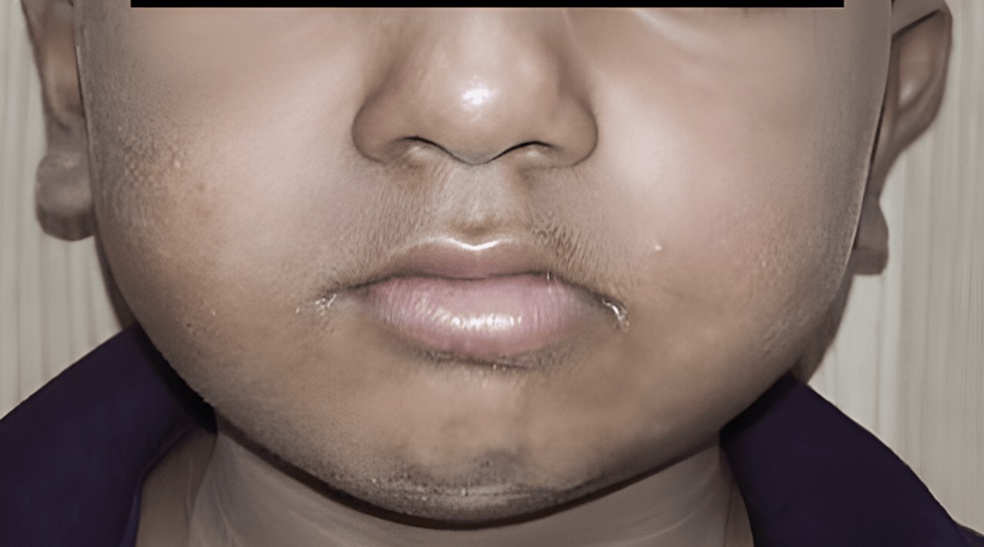
Extra-oral follow-up image.

Postoperative follow-up was done every week for up to two weeks. Healing was uneventful. After 10 days, the elastics given for inter-maxillary fixation (IMF) were removed. The open cap splint was left in situ. The patient was advised to be on a liquid and soft diet along with antibiotics (amoxicillin 125 mg TDS) and analgesics (ibuprofen 100 mg BD) for one week. The patient was given oral hygiene instructions, which included oral rinsing after every meal and supervised brushing. The open cap splint was removed after two months under local anesthesia as the patient did not report to the department after one month. There was no intersegmental mobility of the fractured segment was seen and occlusion was stable bilaterally. The fractured site showed good healing on OPG, and satisfactory occlusion was achieved (Figure 6). The prognosis in this case was good, and the patient was kept on monthly follow-up for up to five months (Figure 7).

## Discussion

Between the juvenile and adult populations, different forms of fracture occur more often. Among the adult population, a fracture of the nasal bone is the most frequent facial fracture, whereas among children mandibular fractures are the most common [6,7]. This is explained by the differential growth patterns of cranial and facial volumes, coupled with the forward and downward expansion of the mandible and midface as age progresses. Because of the highly vascularized pediatric condyle, thin cortices, and significant amount of medullary bone, which are incapable of resisting the impact of fall, condylar fractures (31%-45%) are more prevalent in children than parasymphysis fractures (13%-26%) [8-10].

The age of a child, stage of tooth growth, and type of fracture are the three most crucial factors in deciding how to treat fractures in the case of pediatric patients. The restoration of preinjury occlusion and underlying bone architecture is the technical aim of fracture treatment. When using open reduction and internal fixation on youngsters, it is advised that plates avoid crossing suture lines or the mandibular midline. Therefore, splints are more reliable than open reduction [11]. The advantages of a splint include simplicity of application and removal, quicker and easier splint construction, shorter operating times, maximal stability during the healing process, little stress to nearby anatomical structures, and tooth-to-tooth contact to aid in occlusion-guided reduction. Advantages of occlusal splints with circum-mandibular wiring in displaced fractures include their usage in a wide range of age groups, early mobilization, and decreased risk of muscular atrophy or ankylosis [5].

Circum-zygomatic fixation along with closed reduction offers a conservative alternative to conventional surgical care in lowering the possibility of postoperative complications. Because of poor retention due to conical-shaped teeth, dental spacing, and partially erupted teeth, IMF with an open reduction to correct occlusion and facial fractures, yields poor results in the primary or mixed dentition and also has the potential for tooth avulsion [12]. Therefore, in this case, a noninvasive approach was taken. For IMF, arch bar hooks were inserted into the open cap splint. Fractures can be reduced and stabilized using a minimally invasive technique while also maximizing healing by making use of the osteogenic potential.

Due to their propensity for osteogenesis, patients less than 16 years old with facial fractures may require early anatomical reduction with brief periods of stabilization. The additionally increased vasculature, oxygenation, and nourishment that a child's more prominent periosteum offers to the osteochondroprogenitor cells increases the likelihood of bone union and remodeling. Pediatric patients experience fewer complications like malunion, nonunion, malocclusion, or postoperative infections because of their increased osteogenic capacity and remodeling. A nonoperative method, however, is not an option when the fracture is complicated with highly movable or displaced segments and associated with orbital dystopia and malocclusion. A minimally invasive approach uses the inherent osteogenic potential of the bone to optimize healing, thereby allowing the reduction and stabilization of fractures [12]. Ensuring satisfactory outcomes on both aesthetic and functional bases necessitates long-term follow-up in the case of pediatric patients with facial fractures [2]. The current case report demonstrates that cap splints are a viable fixing method in terms of occlusion-guided fracture reduction and maximal stability during the healing period.

Cap splints are a versatile treatment option for juvenile mandibular fractures because they can be used to restore function and aesthetics with minimal morbidity, do not impede jaw growth or the development of dentition, and can be applied to patients of a wider range of ages [1]. On a dental model, a retention-modified, occlusal splint was made in this case. Closed reduction with circum-zygomatic and circum-mandibular wiring held in place for two months by the occlusal splint before being removed. The patient exhibited a good facial profile, symmetry, and function at the three-month review. The outstanding cosmetic and functional results might be achieved without the possible morbidity associated with a more conservative approach. The purpose of this case report is to provide insight into pediatric maxillofacial injuries and assist the operator in conservatively managing maxillary Le Fort and mandibular symphysis fractures in children with the help of a cap splint as a definitive treatment option. This is an alternative technique in the pediatric population to open reduction with internal fixation of complex mandibular and midface fractures.

## Conclusions

While treating mildly displaced fractures, a conservative approach is preferable. Anatomical reduction of facial fractures along with restoration of facial symmetry, cosmesis, and function can be achieved by circum-zygomatic and circum-mandibular closed reduction accompanied by retention modified occlusal splinting. In the pediatric population, this method should be taken into consideration as an alternative to open reduction internal fixation for displaced complicated midface fractures.
